# Symptoms and joint degeneration correlate with the temperature of osteoarthritic knees: an infrared thermography analysis

**DOI:** 10.1007/s00264-024-06376-1

**Published:** 2024-11-26

**Authors:** Luca De Marziani, Lorenzo Zanasi, Giacomo Roveda, Angelo Boffa, Luca Andriolo, Alessandro Di Martino, Stefano Zaffagnini, Giuseppe Filardo

**Affiliations:** 1 Clinica Ortopedica e Traumatologica 2, IRCCS Istituto Ortopedico Rizzoli, Via Giulio Cesare Pupilli, 1, Bologna, 40136 Italy; 2 Applied and Translational Research (ATR) Center, IRCCS Istituto Ortopedico Rizzoli, Bologna, Italy

**Keywords:** Symptoms, Joint degeneration, Knee, Inflammation, Thermography

## Abstract

**Purpose:**

This study aim was to analyze the joint temperature of patients affected by bilateral knee osteoarthritis (OA) using infrared thermography to investigate whether thermographic imaging patterns are influenced by the severity of symptoms and joint degeneration.

**Methods:**

Sixty-sixpatients ranging from 43 to 78 years old (63.3 ± 8.8 years) with bilateral knee OA and one symptomatic knee were enrolled. Thermograms of the two knees were captured using a thermographic camera FLIR T1020 and analyzed with the ResearchIR software to calculate the temperature of the overall knee and the four regions of interest (ROIs): patella, suprapatellar, medial, and lateral areas.

**Results:**

The temperature of knees affected by OA was influenced by joint degeneration level and symptoms: patients with higher OA grade in the symptomatic knees presented higher total knee temperatures compared to the asymptomatic ones (*p* = 0.002), as well as in the patellar (*p* = 0.005), lateral (*p* = 0.002), and medial (*p* = 0.001) areas. On the other hand, patients with the same OA level in the two knees presented a higher temperature in the symptomatic knee only in the medial area (*p* = 0.037). Symptomatic knees demonstrated a different pattern compared to asymptomatic knees, with the medial area presenting the highest temperature changes (*p* = 0.020). Patients reporting prevalent pain in the lateral knee area presented higher differences in total knee temperature (0.7 ± 0.7 °C) than patients with pain in the medial area (0.1 ± 0.5 °C) (*p* = 0.023).

**Conclusion:**

The temperature of knees affected by OA is influenced by the degree of joint degeneration and by the presence of symptoms, with higher temperatures found in symptomatic joints, especially with prevalent lateral knee pain, and in more severe OA. Symptomatic knees demonstrated a different pattern compared to asymptomatic knees, with the medial area presenting the highest temperature changes.

## Introduction

Infrared thermography is a method to quantify the infrared radiation emitted from a defined body region, converting it into temperature data and producing a digital image based on a colour scale [1]. This technology can identify thermal abnormalities by detecting changes in skin surface temperature, which may indicate the presence of underlying pathologies [2]. More in detail, infrared thermography has been proposed as a method for quantifying and monitoring inflammation, which is a key aspect of the pathophysiology of several diseases [3]. The awareness of the role of inflammation in a wide range of diseases, as well as improvements in thermal imaging both in terms of cameras and advanced software for image analysis, led to an increased use of infrared thermography in several fields [1]. In recent years, infrared thermography has been explored in the orthopedic field to study patients with knee osteoarthritis (OA), improve knowledge on the pathology, and guide patient-specific treatments [4].

Knee OA is a common disease of the joint affecting more than 20% of patients over 40 years old [5]. It is characterized by wear and degeneration of the articular cartilage with concomitant structural and functional changes across the entire joint [6]. These degenerative changes are associated by a proinflammatory environment with the release of inflammatory cytokines and synovial inflammation resulting in synovial hyperplasia, angiogenesis, and effusion [7, 8]. Infrared thermography can study the joint inflammation that characterizes OA knees through the evaluation of the skin temperature [3, 7, 9]. However, while its use is increasing in this field, a lack of evidence remains on factors that may influence the evaluation of knee OA in the clinical practice [9–11]. Preliminary studies suggested that knee temperature is influenced by the severity of symptoms and joint degeneration, but these studies compared mono-lateral OA joints of different patients, with biases related to the heterogeneous patients’ characteristics that impair drawing clear conclusions, and bilateral evaluations with intra-patient controls are still lacking to guide the interpretation of joint temperature in knee OA [9, 12].

The aim of this study was to analyze the joint temperature of patients affected by bilateral knee OA using infrared thermography to investigate whether imaging patterns are influenced by the severity of symptoms and joint degeneration.

## Materials and methods

This study was approved by the hospital Ethics Committee of the IRCCS Istituto Ortopedico Rizzoli, Italy (approval number 0017413) in accordance with the declaration of Helsinki. Orthopedic physicians enrolled patients between December 2021 and April 2024 in a research outpatient clinic specialized in knee OA. Informed consent was obtained from each patient before the study participation. The study included male and female patients with bilateral knee OA (Kellgren–Lawrence– KL – grade ≥ 2) and with one symptomatic knee (Visual Analogue Scale – VAS – for pain ≥ 2) for at least six months and the contralateral knee asymptomatic (VAS for pain = 0). Exclusion criteria included: History of trauma or intra-articular injection therapy within six months before to thermographic evaluation, knee surgery within the past twelve months, any concomitant knee condition causing pain or swelling, systemic disorders such as thyroid metabolic disorders, uncontrolled diabetes, dermatological and vascular conditions, inflammatory arthropathy, severe cardiovascular diseases, hematological diseases, infections, immunosuppression, neoplasms, antidepressant, anticoagulant, or antiaggregant therapy, and the use of nonsteroidal anti-inflammatory drugs within five days before the investigation.

Sixty-six patients were enrolled according to the inclusion/exclusion criteria. Among them, 48 patients were men and 18 women, with a mean age of 63.3 ± 8.8 years and a mean body mass index (BMI) of 25.4 ± 3.2. Patients’ characteristics are reported in detail in Table 1. The symptomatic knees had a mean VAS pain of 5.7 ± 2.0 and an International Knee Documentation Committee (IKDC) objective score rated as A in 11 patients, B in 31, C in 14 and D in 10. On the other side, the asymptomatic knees had a VAS pain of 0.0 ± 0.0 and an IKDC objective score rated as A in all patients.

**Table 1:** Patients’ characteristics

Sex (M/W)	48/18
Age (years)	63.3 ± 8.8
BMI (Kg/m^2^)	25.4 ± 3.2
Smoke (yes/no)	8/58
Side symptomatic knee (left/right)	27/39
Symptomatic knee KL grade	Grade 2: 5
	Grade 3: 56
	Grade 4: 5
Symptomatic knee KL grade (mean)	2.5 ± 0.6
Asymptomatic knee KL grade	Grade 2: 34
	Grade 3: 29
	Grade 4: 3
Asymptomatic knee KL grade (mean)	2.0 ± 0.4

Values are expressed as mean ± standard deviation. BMI, body mass index; KL, Kellgren-Lawrence; M, men; W, women

After the enrollment, patients were clinically evaluated for their symptomatic knees thorough knee-specific patient reported outcome measurements (PROMs) including the International Knee Documentation Committee (IKDC) subjective and objective scores, the Knee injury and Osteoarthritis Outcome Score (KOOS) sub-scales, VAS for the symptomatic knee pain, the PainDETECT Questionnaire for the neuropathic pain evaluation, and the Tegner activity level score. Patients compiled the subjective clinical questionnaires with the clinician support, while the IKDC objective score was evaluated by the clinician. The clinical characteristics of the symptomatic knees are reported in Table 2.

All participants underwent weight-bearing antero-posterior and a lateral X-ray of the knee to assess the baseline OA severity according to the KL grading system [13]. The symptomatic knees presented a KL grade higher compared to the asymptomatic knees (*p* < 0.0005), with 31 patients having a higher KL grade in the symptomatic knee and 35 patients having the same KL grade in the two knees.

**Table 2:** Symptomatic knee characteristics

Symptoms duration (months)	102.8 ± 113.6
VAS pain	5.7 ± 2.0
Prevalent knee pain localization	Anterior: 9
	Medial: 34
	Lateral: 16
	Widespread: 7
IKDC Subjective score	42.7 ± 13.8
IKDC Objective score	Grade A: 11 Grade B: 31 Grade C: 14 Grade D: 10
KOOS Pain	58.5 ± 16.7
KOOS Symptoms	60.0 ± 19.1
KOOS ADL	69.0 ± 17.3
KOOS QoL	33.6 ± 16.9
KOOS Sport/Rec	37.4 ± 22.3
Tegner score	2.2 ± 1.2
PainDETECT Questionnaire	8.5 ± 5.7

Values are expressed as mean ± standard deviation. ADL, activities of daily living, IKDC, International Knee Documentation Committee; KOOS, Knee Injury and Osteoarthritis Outcome Score; QoL, quality of life; Sport/Rec, function in sport and recreation; VAS, visual analogue scale

### Infrared thermography procedure and analysis

The skin temperature of both symptomatic and asymptomatic knees was evaluated thorough infrared thermography. According to the current guidelines [14], patients were asked to respect specific instructions before the infrared thermography procedure: Avoidance of physical activity within 48 h; avoidance of alcohol beverages, smoking, caffeine, any type of cosmetics, and showering within  four h; and avoidance of knee exposure to the sun for long periods during the week prior to the examination.

Infrared imaging evaluation was acquired in a dedicated outpatient clinic with a temperature controlled and set at 23.0 °C, a mean humidity of 45 ± 15%, and shielded from direct sunlight. In order to minimize the circadian variation of the temperature, image acquisition was always performed in the same time slot between 15:00 pm and 18:00 pm. According to previous literature [15], participants were asked to sit for ten min with light clothing on the top to accelerate the thermalization, without touching their knees before image acquisition. The participants were asked to stand on a designated floor map. The thermograms of the two knees were captured using a thermographic camera FLIR T1020 (FLIR^®^ Systems, Stockholm, Sweden), which has 1024 × 768 pixels of resolution and a thermal sensitivity of 0.02 °C. The camera was positioned at 1 m of distance from the subject, positioned perpendicular to the knees, and adjusted to their patellar height. An anterior view image was obtained for each patient using the autofocus modality. Then, maintaining the same knee position, a circular marker (2 cm diameter) was placed at the center of the patella and a second anterior image was obtained to facilitate the precise localization of the patellae in the infrared images [9].

**Fig. 1:**
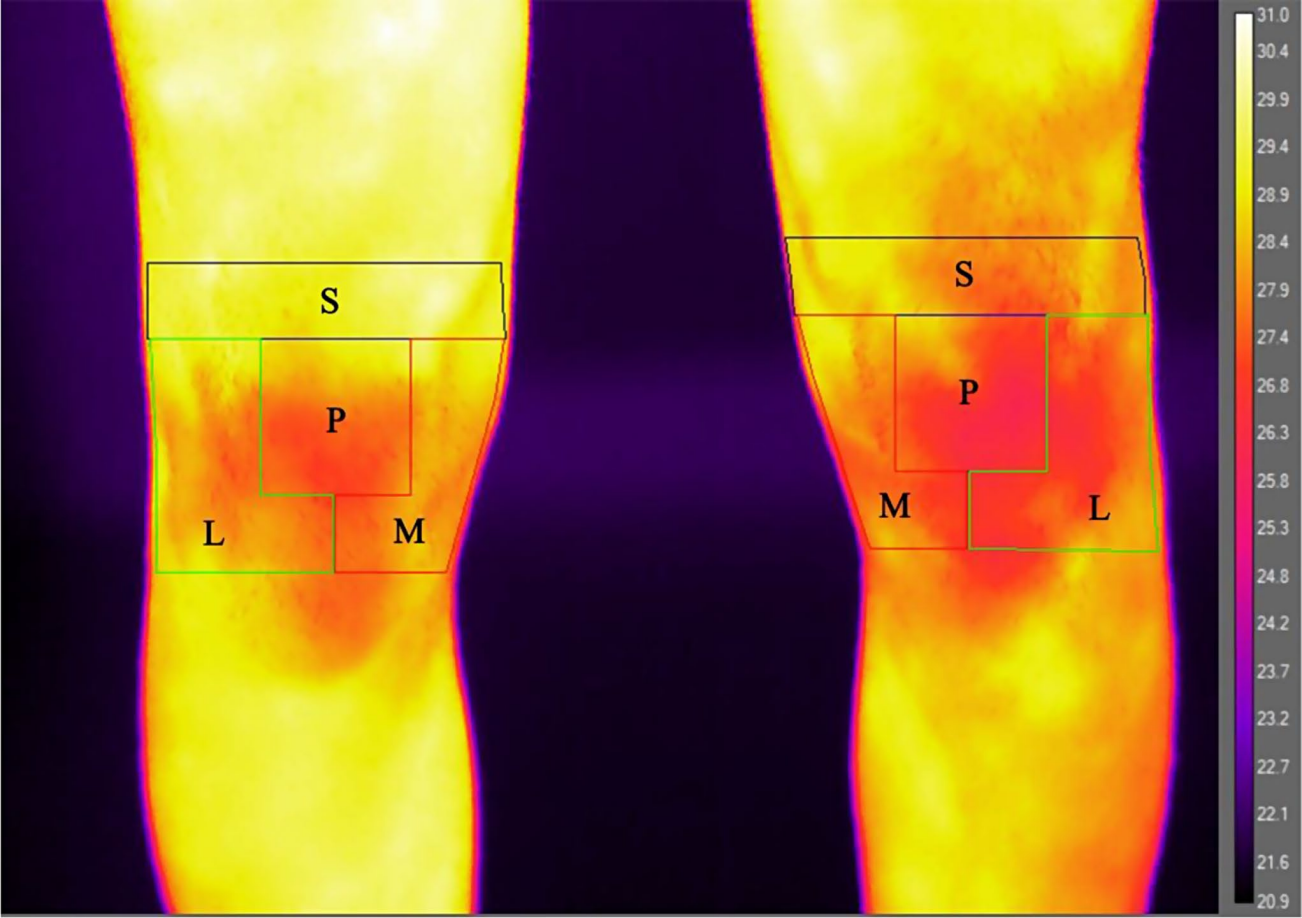
The symptomatic knee (left image) presents a higher temperature compared to the contralateral healthy knee. Both knees were analyzed through the same regions of interest: L, lateral; M, medial; P, patella; S, suprapatellar

For the image analysis process, the anterior images acquired were aligned side by side with the patellar marker image on the computer screen, and, using the marked image as a guide, a template indicating the region of interests (ROIs) was centered over the patella of each unmarked image. The ROIs were defined as follows: “patellar” area - a square of 6 cm in width centered on the patella, “suprapatellar” area - the area 3 cm over the patella; “medial” and “lateral” areas - the regions 3 cm under the patella and on its medial and lateral sides, respectively [9]. The sum of the 4 ROIs was defined as the “total knee temperature”. The mean temperatures were extracted using the software ResearchIR (FLIR^®^ Systems, Sweden) for the overall knee area and for the 4 ROIs: patella, suprapatellar, medial, and lateral. The thermographic images obtained for both the symptomatic and asymptomatic knees were compared to analyze intra-patient differences (Fig. 1). Moreover, correlations between the thermographic images and knee characteristics were investigated.

### Statistical analysis

All continuous data were expressed in terms of mean ± SD, categorical variables were expressed as proportions or percentages. The Shapiro–Wilk test was performed to test normality of continuous variables. The Levene test was used to assess the homoscedasticity of the data. The Paired t test was used to assess the difference between the symptomatic and the asymptomatic knees. The Repeated Measures General Linear Model (GLM) with Sidak test for multiple comparisons was performed to assess the differences among the different areas and as multivariate analysis to investigate the factors influencing the differences between symptomatic and the asymptomatic knees. The Friedman non parametric test, followed by the Wilcoxon post hoc pairwise comparison corrected by Bonferroni method for multiple comparisons, was used to assess the differences in the different areas of ordinal scores. The ANOVA test was performed to assess the between groups differences of continuous, normally distributed and homoscedastic data, the Mann Whitney test was used otherwise. The ANOVA test followed by the Scheffè post hoc pairwise comparison was used also to assess the among groups differences of continuous, normally distributed and homoscedastic data, the Kruskal Wallis test followed by the Mann Whitney test with the Bonferroni correction for multiple comparison was used otherwise. The Spearman rank Correlation was used to assess correlations between numerical scores and continuous data, the Kendall Tau correlation was used to assess correlations between ordinal scores and continuous data. The Pearson Chi square evaluated using exact test was performed to investigate relationships between grouping variables. For all tests *p* < 0.05 was considered significant. All statistical analysis was performed using SPSS v.19.0 (IBM Corp., Armonk, NY, USA).

## Results

### Knee temperature

**Fig. 2:**
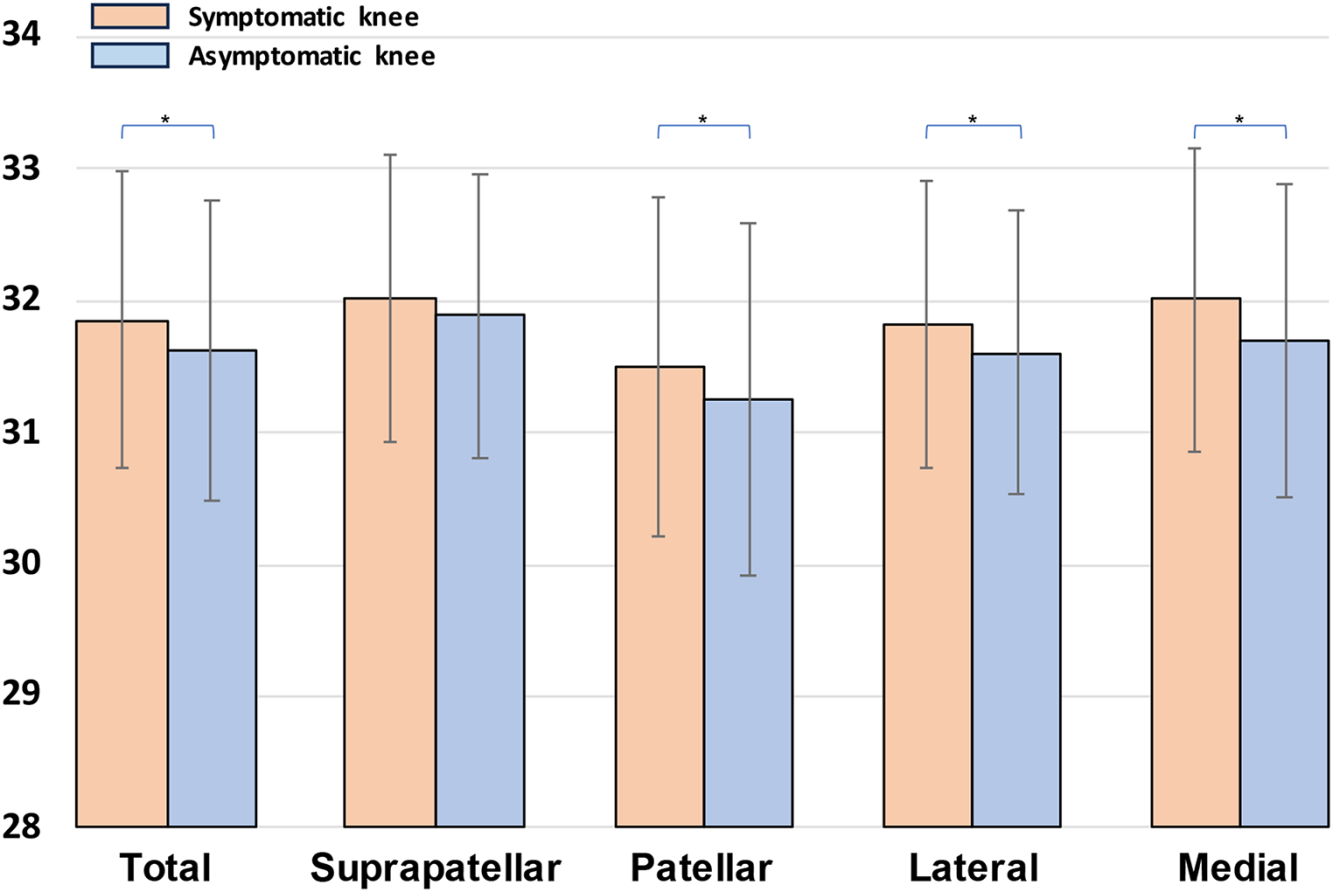
Mean knee temperature of symptomatic and asymptomatic knees for the total knee temperature and all sub-areas. Presented as mean and standard deviation. The orange columns represent the temperature of the symptomatic knees, while the blue columns represent the temperature of the asymptomatic knees. *Significant differences

The mean temperature of the total knee was 31.9 ± 1.1 °C for the symptomatic knees and 31.6 ± 1.1 °C for the asymptomatic knees (Fig. 2). The mean temperature of the total knee was significantly higher for the symptomatic knees compared to the asymptomatic knees (*p* = 0.003). Analyzing the temperature of the different areas, the mean temperature of the symptomatic knees was significantly higher compared to the asymptomatic knees for the patellar (31.5 ± 1.3 °C vs. 31.2 ± 1.3 °C; *p* = 0.015), the lateral (31.8 ± 1.1 °C vs. 31.6 ± 1.1 °C; *p* = 0.016), and the medial (32.0 ± 1.1 °C vs. 31.7 ± 1.2 °C; *p* < 0.0005) areas, while no statistically significant difference was found for the suprapatellar area (32.0 ± 1.1 °C vs. 31.9 ± 1.1 °C; n.s.).

The analysis of the sub-areas demonstrated that the patellar area is the coldest region of the knee compared to the other sub-areas both in the symptomatic and in the asymptomatic knees (all *p* < 0.0005). The suprapatellar area was significantly warmer compared to all other sub-areas in the asymptomatic knees, while in the symptomatic knees this area remained significantly warmer only compared to the patellar (*p* < 0.0005) and lateral area (*p* = 0.015). This different behavior was led by the trend observed for the medial area between the symptomatic and asymptomatic knees. In fact, while in the asymptomatic knees the medial area did not show temperature differences compared to the lateral area (p = n.s.) and had lower temperatures than the suprapatellar area (*p* = 0.005), in the symptomatic knees the medial area showed higher temperatures than the lateral area (*p* = 0.020) and reached similar temperatures to the suprapatellar area (p = n.s.).

### Factors influencing knee temperature

The joint pain localization in symptomatic knees was found to influence the difference in total knee temperature between symptomatic and asymptomatic knees. In detail, patients reporting pain in the lateral knee area presented higher differences in total knee temperature (0.7 ± 0.7 °C) than patients with pain in the medial knee area (0.1 ± 0.5 °C) (*p* = 0.023).

To investigate the influence of the KL grade on knee temperature, patients were divided in two groups: 35 patients with the same KL grade in symptomatic and asymptomatic knees and 31 patients having a higher KL grade in the symptomatic knee. Patients with higher KL grade in the symptomatic knees presented higher total knee temperatures in the symptomatic knees compared to the asymptomatic ones (32.3 ± 1.0 vs. 31.9 ± 1.0, *p* = 0.002), as well as in the patellar (32.0 ± 1.2 vs. 31.6 ± 1.3, *p* = 0.005), lateral (32.3 ± 1.0 vs. 31.8 ± 0.9, *p* = 0.002), and medial (32.4 ± 1.0 vs. 32.0 ± 1.1, *p* = 0.001) areas.

On the other hand, patients with the same KL grade of the two knees presented higher temperatures in the symptomatic knee only in the medial area (31.6 ± 1.1 vs. 31.4 ± 1.2, *p* = 0.037). Thus, the analysis of the differences in knee temperatures between the symptomatic and asymptomatic knees for these two groups showed that the medial knee area was affected by the presence of symptoms, rather than by OA severity (Table 3).

**Table 3:** Knee temperature in patients with different or same Kellgren-Lawrence levels

	Knees with different Kellgren-Lawrence levels			Knees with same Kellgren-Lawrence levels		
	Symptomatic	Asymptomatic	*p*	Symptomatic	Asymptomatic	*p*
Total	32.3 ± 1.0	31.9 ± 1.0	0.002	31.4 ± 1.0	31.4 ± 1.2	n.s.
Suprapatellar	32.4 ± 1.0	32.2 ± 1.0	n.s.	31.7 ± 1.1	31.6 ± 1.1	n.s.
Patellar	32.0 ± 1.2	31.6 ± 1.3	0.005	31.0 ± 1.2	31.0 ± 1.3	n.s.
Lateral	32.3 ± 1.0	31.8 ± 0.9	0.002	31.4 ± 1.0	31.4 ± 1.2	n.s.
Medial	32.4 ± 1.0	32.0 ± 1.1	0.001	31.6 ± 1.1	31.4 ± 1.2	0.037

Values are expressed as mean ± standard deviation

The multivariate analysis demonstrated that the difference in total knee temperature between symptomatic and asymptomatic knees corrected for the difference in KL grade is significantly greater, confirming the influence of symptoms (0.225 95% CI 0.085–0.366 *p* = 0.002).

## Discussion

The main finding of this study is that the temperature of knees affected by OA is influenced by the degree of joint degeneration and by the presence of symptoms, with higher temperatures found in symptomatic joints, especially with prevalent lateral knee pain, and in more severe OA. Symptomatic knees demonstrated a different pattern compared to asymptomatic knees, with the medial area presenting the highest temperature changes.

Evidence on the clinical application of infrared thermography for knee OA is still limited and understanding its clinical role remains challenging, with the main reason lying in the multitude of factors capable of modifying the temperature of OA joints [10, 16]. Previous studies highlighted how several patient characteristics can influence knee temperature. A recent study on more than 900 healthy people established that gender influences the lower limb temperature, with men reporting a temperature about 2 °C higher than women [17]. Another recent study focused on 60 patients with knee OA and confirmed higher temperatures in the knees of male patients [11]. Other studies highlighted how knee temperature in OA patients is influenced by BMI and age, with higher joint temperature in younger patients with higher BMI [9, 18]. However, all these studies evaluated the temperatures of OA knees of different patients, whose several heterogeneous characteristics could make it difficult to determine the exact impact of each characteristic analyzed. This reduces the clinical relevance of previous findings.

This study eliminated the influence of inter-patient variables affecting knee temperature by analyzing patients with bilateral knee OA and documenting asymmetries between the two knees of the same patients. Body temperature tends to thermal symmetry within the same patient [17, 19, 20]. Accordingly, the evaluation of patients with bilateral knee OA allowed to delete the confounding factors related to the comparison of different patients, including age, sex, and BMI, thus ensuring a more reliable evaluation of characteristics specific of the disease that could affect the joint temperature, including the presence of symptoms and OA severity.

This study demonstrated that OA severity can influence knee temperature. Joints with a higher KL grade showed higher temperatures compared to those with lower KL grades, both in the total knee and in the sub-areas. These results confirmed those suggested by previous studies. A study by Denoble at al. [10] on a small cohort of 30 women, 15 affected by mono-lateral knee OA and 15 healthy controls, highlighted how the degree of joint degeneration was correlated to the skin temperature above the patella. Moreover, another study by Lohchab et al. on 62 patients with grade 1–4 knee OA highlighted how the knee temperature increased with the increase of joint degeneration [18]. As confirmed by the current study on bilateral knee OA patients, the degree of joint degeneration is one of the key factors capable of influencing the temperature of OA knees. A possible explanation for the higher temperature in the more degenerated knees could be the abnormal load distribution on the articular surface and cartilage degeneration, which may result in the increased joint inflammation detected by infrared thermography [21].

The higher inflammatory state may lead to increased symptoms [7]. The study results demonstrated higher temperatures in the symptomatic knees of OA patients compared to asymptomatic ones, confirming a direct correlation between knee temperature and symptoms. These results are conflicting with those of a previous study by Vargas et al. [16] on a smaller population of 25 patients showing no differences in the temperature between symptomatic and asymptomatic knees. However, these authors took in consideration only a single ROI of the knee, thus impairing the possibility to develop an accurate characterization of the different knee areas and understand the influence of symptoms in the different joint areas. To this regard, the analysis of patients with the same KL grade produced interesting findings. In fact, while no differences were observed in the overall knee ROI, patients presented a significantly higher temperature in the medial ROI area of the symptomatic knee compared to the asymptomatic one. Higher temperatures in the medial area of symptomatic OA knees were also reported in a previous study by Arfaoui et al. [22] on10 patients with knee OA compared with 12 healthy controls. These findings confirmed the importance to examine the different knee sub-areas for a more complete and reliable evaluation of the temperature in OA knees.

The analysis of the knee sub-areas confirmed an overall higher temperature in the symptomatic knees. The sub-areas characterization also allowed to identify different imaging thermographic patterns between symptomatic and asymptomatic knees, in particular for the medial area. In fact, while medial and lateral areas were comparable in asymptomatic knees, in symptomatic knees the medial area showed higher temperatures. On the other hand, looking at the other sub-areas, no differences were found between symptomatic and asymptomatic knees, and in both knees the area corresponding to the patella was the coldest. This finding is in line with previous literature, which described the thermal image of a healthy knee with a cold isothermal oval area above the patella due to the presence of underlying bone [20, 23–25]. Consequently, the patella represents the coldest area even in patients with knee OA, and this could probably be due to the distance of this area of ​​the knee from the intra-articular environment due to the interposition of the patellar bone. On the contrary, the medial and lateral areas have a greater proximity to the joint environment, thus better reflecting an intra-articular inflammatory state due to increased vascularization of the synovial membrane [26].

Another interesting study finding is related to the sub-analysis on pain knee localization, which demonstrated how the difference in temperature between the symptomatic and asymptomatic knees may be related to where the patient localizes its joint pain. Specifically, patients reporting pain in the lateral area of the knee demonstrated higher differences in knee temperature. Prevalent lateral knee pain may be related to more complex biomechanical reasons, being more frequent in patients with a valgus malalignment of the lower limbs or in patients with previous lateral meniscectomy, with increased loads on the lateral knee compartment [27]. In these patients, the altered load and complex lateral biomechanical environment may lead to more rapid joint inflammation and degeneration, and consequently to a higher knee temperature [28, 29]. However, future studies should better characterize the progression of joint degeneration affecting the external compartment and evaluate how to improve the study and management of these patients also through the use of infrared thermography.

This study presents some limitations. Despite being one of the largest studies on patients with bilateral knee OA, the sample size may limit the possibility to investigate subgroups and identify correlations, and further studies on larger populations are needed to confirm these findings. Moreover, the knee thermal assessments were conducted using only anterior views, which is currently the most used in the literature, but further information could be obtained through lateral and posterior thermal imaging. Another limitation of the study is the lack of specific tests for the evaluation of joint inflammation such as the analysis of inflammatory biomarkers, although the most appropriate biomarkers for this type of analysis have yet to be defined in the literature [30, 31]. Future studies should explore the relationship between inflammatory biomarkers and the temperature of OA knees assessed by infrared thermography. This tool could have the potential to better evaluate knee OA patients, improving diagnosis, management, and treatment. Overall, more data are needed to standardize the use of thermography for the evaluation of patients with knee OA, confirming its potential in identifying various disease patterns in both research and clinical settings. This study confirms previously suggested factors while providing new insights for future research to optimize infrared thermography potential for knee OA.

## Conclusion

This study demonstrated that the temperature of knees affected by OA is influenced by the degree of joint degeneration and by the presence of symptoms, with higher temperatures found in symptomatic joints, especially with prevalent lateral knee pain, and in more severe OA. Symptomatic knees demonstrated a different pattern compared to asymptomatic knees, with the medial area presenting the highest temperature changes.

## Data Availability

The Ethic Committee did not authorize sharing the raw patients’ data. The calculated average values, which protect patients’ privacy, are detailed in the manuscript.
